# Supplementary material for: Insertion of 643bp Retrotransposon Upstream of PPARγ CDS Is Associated with Backfat of Large White Pigs
Source: Animals (Basel). 2023 Jul 19;13(14):2355. doi: 10.3390/ani13142355 (PMC10376311; doi:10.3390/ani13142355)
Supplement: Supplementary file 1 [file animals-13-02355-s001.zip › Supplementary Materials.pdf]

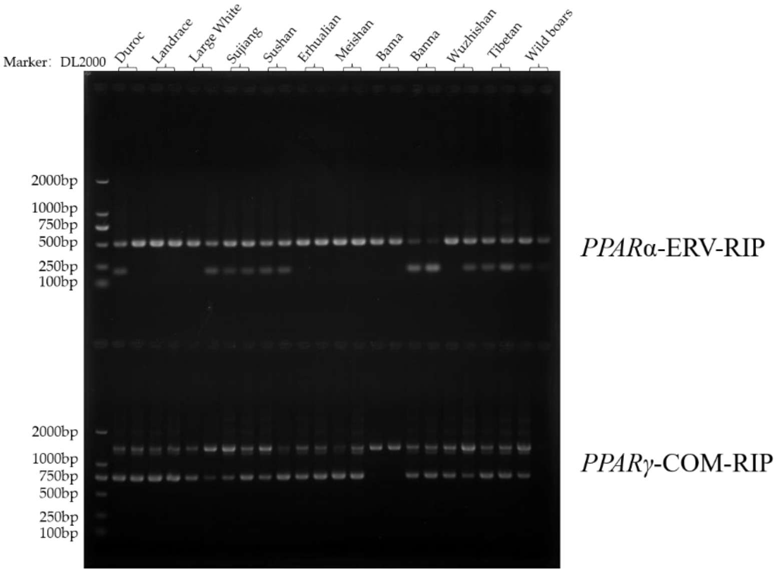

Figure S1. The original electrophoretic figure showing PCR detection of RIPs and genotypes.

Table S1 Number and origin of pig breeds used in RIP detection

Breed	Number	Province/country of origin	Purpose
Duroc	6	Anhui/China	RIP detection
Landrace	6		
Large White	6		
Sujiang	6	Jiangsu/China	
Sushan	6		
Erhualian	6		
Meishan	6		
Bama	6	Guangxi/China	
Banna	6	Jiangsu/China	
Wuzhishan	6	Huainan/China	
Tibetan	6	Sichuan/China	
Wild boars	6	Sichuan/China	

Table S2 *PPARs* gene imformation of other pig breeds or population

Gene	Breed	Serial number	Location	Length /bp
PPARα	Duroc	NC_010447.5	3300755-3323091	22337
	Bama	CM017487.1	104278729-104305065	26337
	Bamei	LUXv01071056.1	1203797-1230340	26544
	Berkshire	LUXw01067681.1	2028367-2054405	26039
	Cross-bred	CM009090.1	103892953-103918210	25258
	Hampshire	LUXS01038505.1	174342-200567	26226
	Jinhua	LUXY01016630.1	265396-291746	26351
	Landrace	LUXT01088799.1	146807-173428	26622
	Large White	LUXx01083335.1	172711-198849	26139
	Meishan	LUXQ01001513.1	227116-253356	26241
	Pietrain	LUxU01003927.1	2258163-2284852	26690
	Rongchang	LUXR01040070.1	6757-33424	26668
	Wuzhishan	KQ004176.1	456943-481882	24940
	Tibetan	AORO02002759.1	1095629-1123119	27491
PPARβ	Duroc	NC_010449.5	31220533- 31297221	76689
	Bama	CM017489.1	67311327-67408919	97593
	Bamei	LUXV01023731.1	128674-211416	82742
	Berkshire	LUXW01024476.1	1771203-1853993	82790
	Cross-bred	CM009092.1	91959191-92041808	82617
	Hampshire	LUXS01012911.1	1800137-1882728	82591

	Jinhua	LUXY01077586.1	2323900-2406516	82616
	Landrace	LUXT01050959.1	9931496-10014210	82714
	Large White	LUXX01073936.1	391211-473727	82516
	Meishan	LUXQ01022153.1	3242663-3325399	82736
	Pietrain	LUXU01004625.1	4401012-4483760	82748
	Rongchang	LUXR01119167.1	1300832-1383450	82618
	Wuzhishan	KQ004112.1	6948236-7032582	84346
	Tibetan	AORO02037490.1	558949-641888	82939
PPARγ	Duroc	NC_010455.5	68299566-68435951	136386
	Bama	CM017495.1	13931504-14075531	144028
	Bamei	LUXV01013805.1	579857-723195	143339
	Berkshire	LUXW01030398.1	546449-690351	143903
	Cross-bred	CM009098.1	138307597-138449948	142352
	Hampshire	LUXS01067119.1	489380-632685	143306
	Jinhua	LUXY01026695.1	579949-722978	143030
	Landrace	LUXT01007590.1	580439-724259	143803
	Large White	LUXX01099939.1	626-131116	130491
	Meishan	LUXQ01098390.1	1631728-1774866	143139
	Pietrain	LUXU01049573.1	3890392-4033636	143245
	Rongchang	LUXR01078567.1	1033855-1177739	143885
	Wuzhishan	AORO02072035.1	37431-182664	145234

Tibetan	KQ003967.1	3712579-3856587	144009

Table S3 The Primers for PCR, vectors construction and q-PCR

Name	Forward primer	Reverse primer	Purpose
PPARα-ERV-RIP	AACGTGCCTTTCATAGTCCA	ACACGTAGAACAATGTCGAG	RIPs identification
PPARγ-SINE-RIP	TGAGCAGCTACAGTATACCAC	ACAGAAATGTGAACACGAGA	
PPARα	GAGTTCGCCAAGTCCATCCC	TGAAGCCATTGCCGTAAGCC	q-PCR
PPARγ	ACATCCCATTCCCGAGAGC	TTTATCCCCACAGACACGGCATT	
ACTB	GCAAGGACCTCTACGCCAAC	TACTCCTGCTTGCTGATCCAC	
PPARγ$^{COM+/-}$-Luc+(EN)	CgacggctcgGCCTGGGAACCTGTATATGGGT	CggaattccgTAGGGGTCTAATCGGAGCTGTAGC	vectors construction

Table S4 Large structural variations (SVs) predicted by alignment in *PPARs* genes and their flanking regions.

	No. of Large SVs			
	Total	*PPARα*	*PPARβ*	*PPARγ*
SVs≥51bp	58	14	19	25
Predicted RIPs	13	4	4	5
Confirmed RIPs	2	1	0	1
